# An ependymin-related blue carotenoprotein decorates marine blue sponge

**DOI:** 10.1016/j.jbc.2023.105110

**Published:** 2023-07-28

**Authors:** Shinji Kawasaki, Takayuki Kaneko, Tomomi Asano, Takashi Maoka, Shinichi Takaichi, Yasuhito Shomura

**Affiliations:** 1 Department of Molecular Microbiology, Tokyo University of Agriculture, Tokyo, Japan; 2 Research Institute for Production Development, Kyoto, Japan; 3 Institute of Quantum Beam Science, Graduate School of Science and Engineering, Ibaraki University, Hitachi, Ibaraki, Japan

**Keywords:** carotenoid, carotenoprotein, X-ray crystallography, lipid-binding protein, protein secretion

## Abstract

Marine animals display diverse vibrant colors, but the mechanisms underlying their specific coloration remain to be clarified. Blue coloration is known to be achieved through a bathochromic shift of the orange carotenoid astaxanthin (AXT) by the crustacean protein crustacyanin, but other examples have not yet been well investigated. Here, we identified an ependymin (EPD)-related water-soluble blue carotenoprotein responsible for the specific coloration of the marine blue sponge *Haliclona* sp. EPD was originally identified in the fish brain as a protein involved in memory consolidation and neuronal regeneration. The purified blue protein, designated as EPD-related blue carotenoprotein-1, was identified as a secreted glycoprotein. We show that it consists of a heterodimer that binds orange AXT and mytiloxanthin and exhibits a bathochromic shift. Our crystal structure analysis of the natively purified EPD-related blue carotenoprotein-1 revealed that these two carotenoids are specifically bound to the heterodimer interface, where the polyene chains are aligned in parallel to each other like in β-crustacyanin, although the two proteins are evolutionary and structurally unrelated. Furthermore, using reconstitution assays, we found that incomplete bathochromic shifts occurred when the protein bound to only AXT or mytiloxanthin. Taken together, we identified an EPD in a basal metazoan as a blue protein that decorates the sponge body by binding specific structurally unrelated carotenoids.

Marine life displays a variety of striking colors, some of which depend on hydrophobic compounds known as carotenoids (1, 2). Certain carotenoids bind to proteins called carotenoproteins. The most well-studied carotenoproteins in marine animals are crustacyanins, which are present in crustaceans. A crustacyanin was first purified from a lobster in 1965 (3). Crustacyanins are nonglycosylated water-soluble blue proteins that are responsible for the blue coloration of crustacean shells. Structural analyses of a crustacyanin in the lipocalin family (4) revealed the presence of noncovalently bound astaxanthin (AXT) in the heterodimer subunits. Carotenoproteins have been detected in various marine animals, including actomyosin in salmon (5), asteriarubin in starfish (6), and ovorubin in the eggs of gastropod snails (7). These carotenoproteins, including crustacyanins, are tightly fixed in the bodies or muscle tissues (1). Although crustacyanins have been extensively studied to elucidate details of the protein structure and the mechanism of the carotenoids’ bathochromic shift (4), relatively little is known about carotenoproteins in other marine species.

Marine sponges are basal metazoans belonging to the phylum Porifera. Similar to other marine organisms, marine sponges display a variety of vibrant colors owing to the presence of carotenoids (8). However, the mechanisms underlying this coloration scheme remain unclear. To the best of our knowledge, four carotenoproteins have been isolated from marine sponges so far (9, 10, 11). A water-soluble blue carotenoprotein (BCP) was isolated from the blue sponge *Suberites domuncula* (9). It binds to the monohydroxy and monoepoxy carotenoids with an absorption maximum at 590 nm. A water-insoluble carotenoprotein was purified from an orange sponge, *Axinella verrucose* (10). However, its primary structures, including the amino acid sequences and their encoding genes, have not been determined.

Carotenoids are hydrophobic and are generally located in lipid globules and cell membranes (1). Examples of protein-bound and water-soluble carotenoproteins include AXT-binding crustacyanin in crustaceans (4, 12, 13), lutein- and zeaxanthin-binding glutathione-*S*-transferase like protein (GSTP1) in the human eye (14), lutein-binding protein in silkworm (15, 16), cyanobacterial orange carotenoid protein (17), and AXT-binding carotenoproteins (AstaPs) in eukaryotic microalgae (18, 19). Although these proteins share a common feature of carotenoid binding, each protein belongs to an individual protein family and is phylogenetically unrelated to the others.

Our recent study found that water-soluble carotenoproteins are present in marine organisms. In the present study, we purified and characterized the blue protein responsible for the coloration of the marine blue sponge, *Haliclona* sp. To the best of our knowledge, the purified protein is the first reported carotenoid-binding protein in the ependymin (EPD) protein family. EPD was first discovered in the ependymal zone of goldfish brain following its enhanced expression after learning events (20, 21, 22). Since then, EPD orthologs known as EPD-related proteins (EPDRs) have been identified in a variety of organisms ranging from basal metazoans to humans (23). Although the roles of EPDs are largely unknown, several studies have reported their involvement in memory consolidation and learning (22, 24, 25), optic nerve regeneration (26), and human brown fat cell development (27). Recent crystallographic studies examined EPDR1, a member of the mammalian EPDR (mammalian EPD-related protein) family (28, 29). They found an antiparallel β-sheet forming a deep hydrophobic pocket, with a possible function to accommodate lipids. Here, we describe the identification and the crystal structure analysis of EPD-BCP1, a novel BCP belonging to the EPD protein family. The characterization of the purified protein revealed that the protein is a heterodimer that accommodates two carotenoids, AXT and mytiloxanthin (MXT). In contrast to the assumption based on the structural studies of EPDR1, our structure analysis of EPD-BCP1 reveals that the two carotenoids are bound to the interface of the heterodimer, rather than the hydrophobic pocket of the β-sheets. Based on the structure and amino acid sequence analyses, the potential mechanisms of the bathochromic shift and the phylogenetic lineage of this protein are discussed.

## Results

### Characterization of sponge samples

**Figure 1:**
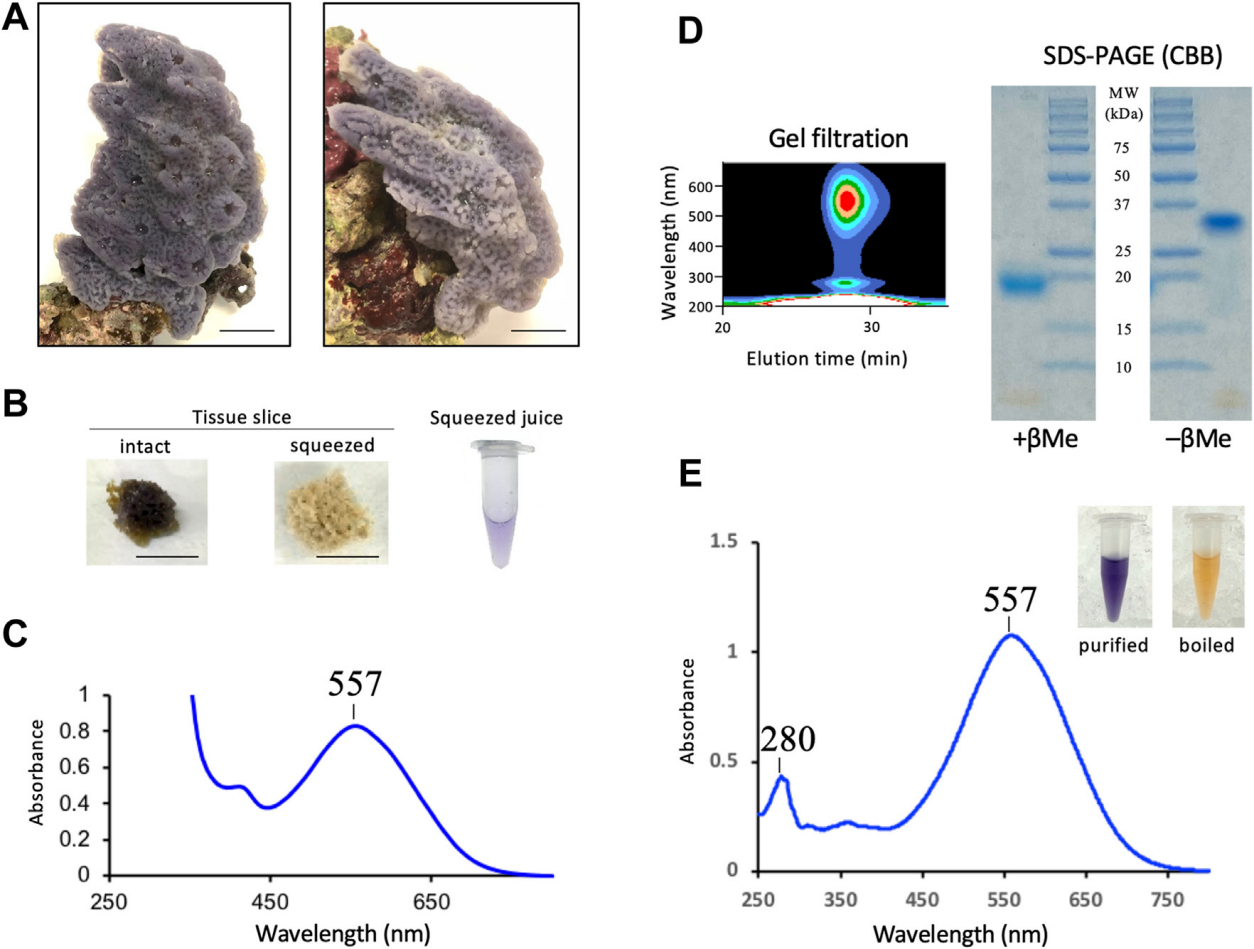
**Purification of blue protein from blue sponges.***A*, photographs of blue sponges collected in 2018 (*left panel*) and 2016 (*right panel*). Scale bar represents 3.0 cm. *B*, change in body color before (*left panel*) and after (*right panel*) squeezing crude extracts from the sponge body. Scale bar represents 1.0 cm. *C*, absorption spectrum of crude extract obtained from a blue sponge collected in 2018. *D*, single elution peak detected with a photodiode array detector following gel filtration (*left panel*). SDS-PAGE of purified blue protein (*right panel*). *E*, absorption spectrum of the purified *blue* protein in Tris–HCl buffer (pH 7.5). Color of the purified protein is shown in the *inset*.

The marine blue sponges used in this study were collected from the reefs offshore of Okinawa prefecture in Japan (Figs. 1*A* and S1). The sponge samples were taxonomically characterized based on morphology (30, 31) and the sequence encoding cytochrome oxidase subunit 1 (cytochrome *c* oxidase subunit 1 [COX1]) (32, 33) with an expert sponge taxonomist. The *cox1* gene sequences of the samples showed homology with other validated Porifera species including *Haliclona amboinensis* (accession no.: KX894495, 100% identity) (33), *Hemigellius pilosus* (accession no.: LN850186, 95% identity) (34), and *Gelliodes wilsoni* (accession no.: KY565314, 91% identity) (35). Moreover, each blue sponge exhibited morphological characteristics similar to the genus *Haliclona*, including spicule morphotypes, ladder-like choanosomal skeleton, and unispicular secondary line (30, 31, 33). Based on morphological and phylogenetic data, the sponge samples were identified as *Haliclona* sp. (Figs. S1 and S2).

### Identification of protein and pigments

**Figure 2:**
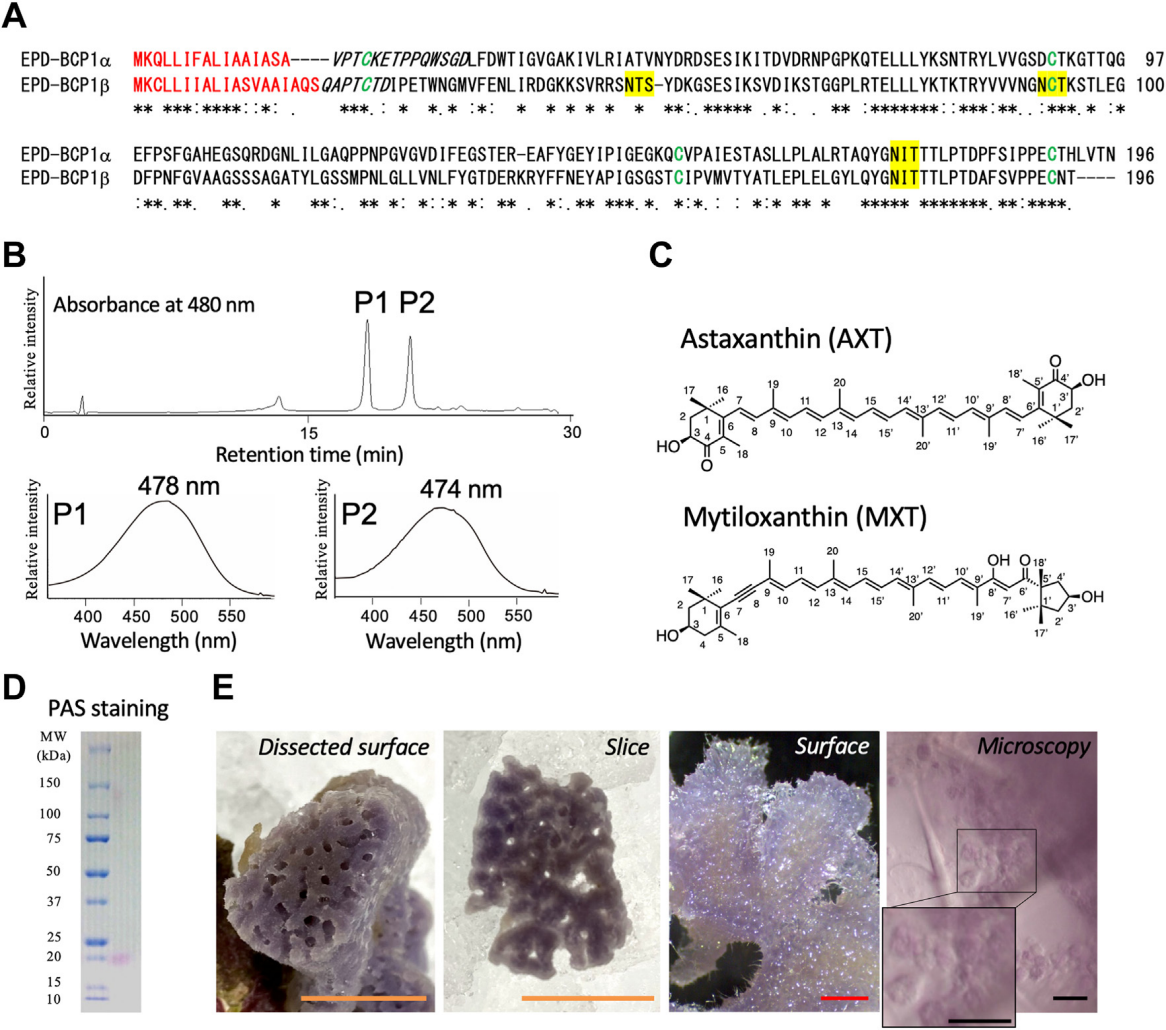
**Primary structure, carotenoid determination, and localization of the purified blue protein.***A*, alignment of deduced amino acid sequences of EPD-BCP1α and EPD-BCP1β. Protein sequences were aligned using ClustalW. Predicted N-terminal signal sequence detected by SignalP is shown in *red*. N-terminal amino acid sequence obtained from purified blue protein is shown in *italics*. Putative N-glycosylation sites are highlighted in *yellow*. Four conserved cysteine residues are shown in *green*. Identical amino acid residues are indicated by *asterisks*, and similar amino acid residues are indicated by *dots*. *B*, HPLC elution profiles of bound carotenoids (*upper panel*) and absorption spectra (*lower panel*) of the peaks P1 and P2. *C*, structures of (3*R*,3′*R*)-astaxanthin (AXT) and mytiloxanthin (MXT). *D*, PAS staining of purified blue protein for detecting protein glycosylation. *E*, localization of *blue* pigment. Dissected surface, a sliced specimen, magnified surface, and microscopic observation of sponge blue cells around the skeleton (*inset*: magnification of the cells). Scale bar represents 1 cm (*orange*), 1 mm (*red*), and 25 μm (*black*). BCP1, blue carotenoprotein-1; EPD, ependymin; Periodic acid–Schiff.

The blue extract was collected by manually squeezing the sponge body. The color of the freshly squeezed extract was the same as that of the body, and the body turned faint brown after squeezing out the extract (Fig. 1*B*). The blue aqueous supernatant was subjected to a gel-filtration column chromatography, from which a single peak representing the blue fraction was obtained (Figs. 1, *C* and *D* and S3). Separation of the purified blue protein by SDS-PAGE under reducing and nonreducing conditions revealed a single band with an apparent molecular mass of 19 kDa and 35 kDa, respectively (Figs. 1*D* and S3). Based on the retention time in gel filtration, the apparent molecular mass of the native protein was estimated at 40 kDa, indicating that the blue protein is a dimer. The purified protein showed absorption maxima at 280 and 557 nm. The broad absorption maximum at 557 nm coincided with that of the crude extract from the sponge body (Figs. 1, *C* and *E*, and S3), indicating that the blue protein constitutes the primary color of the blue sponge. The pigments bound to the blue protein were extracted using the Bligh–Dyer method (36). The organic phase was orange. Separation of the organic phase by HPLC on a C_18_ reversed-phase column yielded two peaks, P1 and P2 (Figs. 2*B* and S3). The compound corresponding to each peak was analyzed by LC–MS. The major component corresponding to P1 exhibited a broad absorption maximum at 478 nm (Figs. 2*B* and S3), and its predicted formula was C_40_H_52_O_4_ ([M + H]^+^ at *m/z* = 597.3941, error = 0.0002), and it had the same retention time as that of the AXT standard (18, 19). The ^1^H NMR spectrum was also compatible with that of standard AXT (Table S1). The chirality of P1 was determined to be (3*S*,3′*S*)-AXT using a Sumichiral OA-2000 column (Fig. 2*C*). The compound corresponding to P2 exhibited a broad absorption maximum at 474 nm, its predicted formula was C_40_H_54_O_4_ ([M + H]^+^ at *m/z* = 599.4095, error = 0.0001), and the ^1^H NMR spectrum was compatible with that of MXT (Table S2) (37, 38, 39). Consequently, the compound corresponding to P2 was identified as MXT (Fig. 2*C*).

### The blue protein belongs to the EPD family

The blue proteins purified from the samples collected between 2016 and 2021 showed similar absorption peaks, molecular weights, and carotenoid compositions. The N-terminal amino acid sequences of the purified blue proteins were VPTXKETPPQWSGD (obtained from the band around 19 kDa) and QAPTXTD (obtained from the band around 20 kDa) (Fig. 2*A*). The genes encoding these N-terminal amino acid sequences were detected in the *de novo* sequencing data of the complementary DNA (cDNA) libraries, and the full-length cDNAs were obtained by PCR. The amino acid sequences deduced from the cDNAs conserved N-terminal hydrophobic signal sequences and putative N-glycosylation (Asn-x-Thr) sites, and the two proteins showed 50% amino acid sequence identity to each other (Fig. 2*A*). A putative EPD domain (accession no.: pfam00811) was detected through a BLAST search. The blastp top hit was a protein with unknown function encoded in the genome of the demosponge *Amphimedon queenslandica* (40) (accession no.: XP_003389285, 39% identity). An N-terminal hydrophobic peptide was detected for each protein using the SignalP program, and the cleavage sites were determined by N-terminal amino acid sequencing between Ala16 and Val17 (in EPD-BCP1α) and between Ser19 and Gln20 (in EPD-BCP1β) (Fig. 2*A*). Periodic acid–Schiff staining revealed that the purified protein was glycosylated (Fig. 2*D*). These results suggest that the purified blue protein is located outside the plasma membrane like other EPD family proteins (41). Microscopy analyses showed that the blue pigment was localized around the skeletons (Fig. 2*E*). EPD-BCP1 conserved four cysteine residues that are commonly conserved in EPD family proteins and are involved in disulfide-bond formation causing dimerization (Figs. 2*A* and 6*B*). We designated this protein as EPD-BCP1 (EPD-related AXT- and MXT-binding BCP1), with its subunits abbreviated as EPD-BCP1α and EPD-BCP1β, based on its pigment-binding properties and the homology search results.

**Figure 3:**
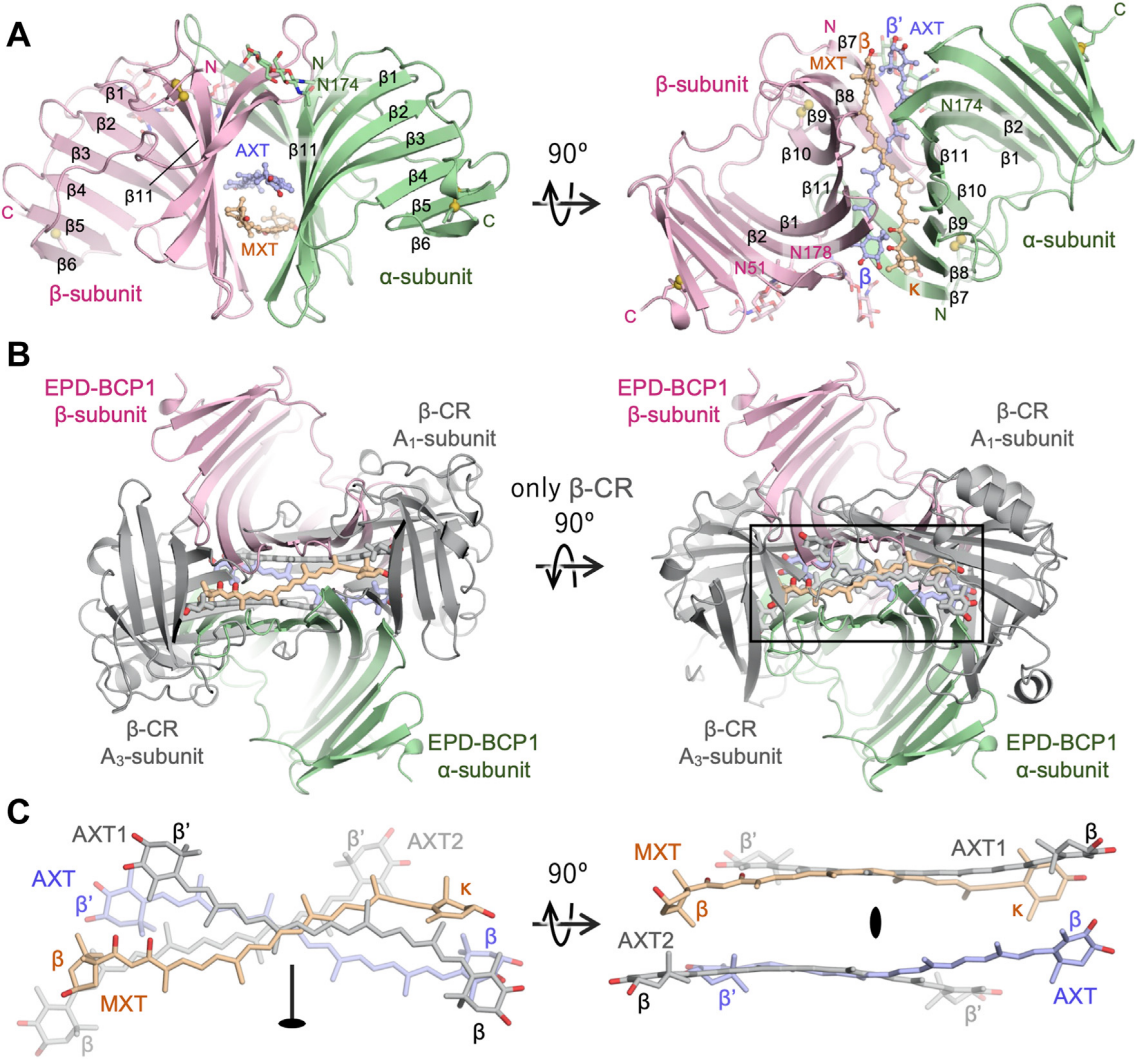
**Crystal structure of EPD-BCP.***A*, overall structure of the heterodimer, with α- and β-subunits shown in different colors. N and C termini are labeled as N and C, respectively. AXT and MXT are shown with the *ball-and-stick model* and labeled. N-glycans and the linking asparagine residues are shown with the *stick model* and labeled. Pairs of cysteine residues forming disulfide bonds are shown with the *ball-and-stick model*. *B*, EPD-BCP1 and β-CR are superposed in a way that the pseudo-twofold axis of each heterodimer is in the center of the figure and perpendicular to the paper. In the *right panel*, only β-CR is rotated by 90° clockwise along the *x*-axis. *C*, comparison of pigment configuration between EPD-BCP1 and β-CR (PDB ID: 1GKA). *Left panel* is the view of the rectangular window in the *right panel* of *B*. Two AXT molecules observed in β-CR are labeled as AXT1 and AXT2 as in Ref. (4). The pseudo-twofold axis found in AXT1–AXT2 in β-CR is shown. β-CR, β-crustacyanin; AXT, astaxanthin; BCP, blue carotenoprotein; EPD, ependymin; MXT, mytiloxanthin.

**Figure 4:**
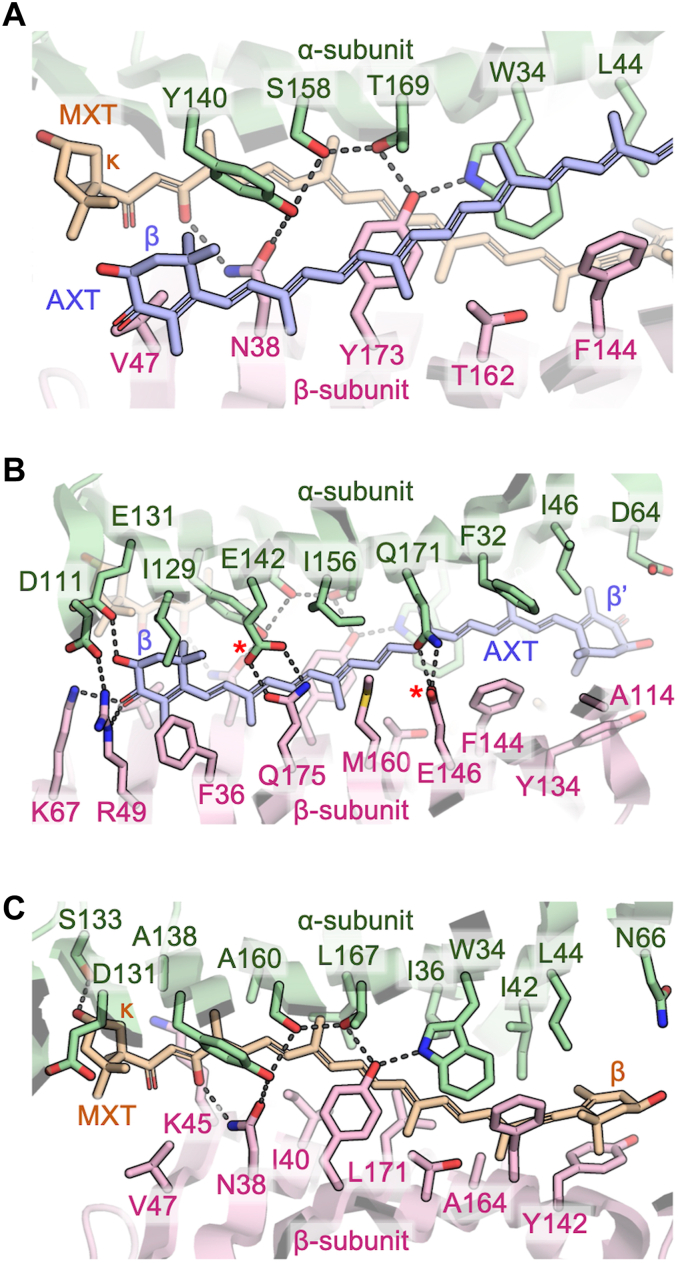
**Protein environment surrounding two carotenoids.***A*, residues in between AXT and MXT. The aromatic ring of F144(β) exceptionally protrudes over AXT. *B*, residues within 4 Å of AXT. The atoms in carboxylate groups labeled with *red asterisks* are assumed to be protonated. *C*, residues within 4 Å of MXT. AXT is omitted for clarity. In *B* and *C*, some labels displayed in *A* are also omitted for clarity. In *A*–*C*, hydrogen bonds are shown with *gray dotted lines*. Diagonally positioned residues (*e.g.*, L44(α) and V47(β)) are equivalent to each other. AXT, astaxanthin; MXT, mytiloxanthin.

**Figure 5:**
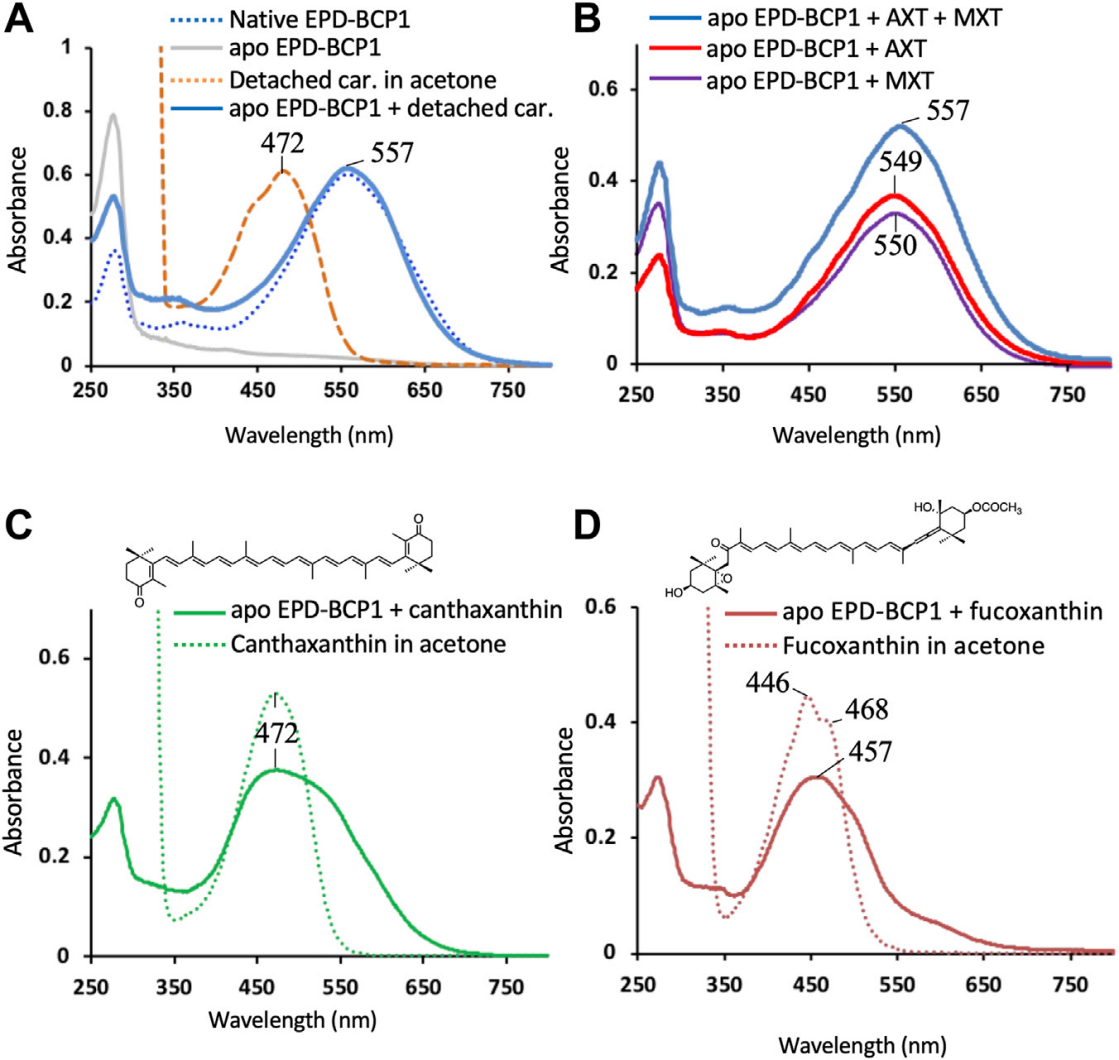
**Reconstitution experiments.***A*, reconstitution of apoprotein with detached *orange carotenoids* (detached car). Spectra of the reconstituted protein (*blue line*) obtained after mixing the apoprotein (*gray line*) with the detached orange carotenoids dissolved in acetone (*orange dotted line*); purified protein used in the reconstitution study (*blue dotted line*). *B*, spectra of holo-EPD-BCP1 reconstituted with astaxanthin (AXT) and mytiloxanthin (MXT) (*blue line*), AXT only (*red line*), and MXT only (*purple line*). *C*, absorption spectrum of holo-EPD-BCP1 reconstituted with canthaxanthin (*green line*), and canthaxanthin dissolved in acetone (*green dots*). Structure of canthaxanthin is indicated above the graph. *D*, absorption spectrum of holo-EPD-BCP1 reconstituted with fucoxanthin (*brown line*), and fucoxanthin dissolved in acetone (*brown dots*). Blank experiment (without protein) did not produce detectable carotenoids by this method. Structure of fucoxanthin is indicated above the graph. BCP1, blue carotenoprotein-1; EPD, ependymin.

**Figure 6:**
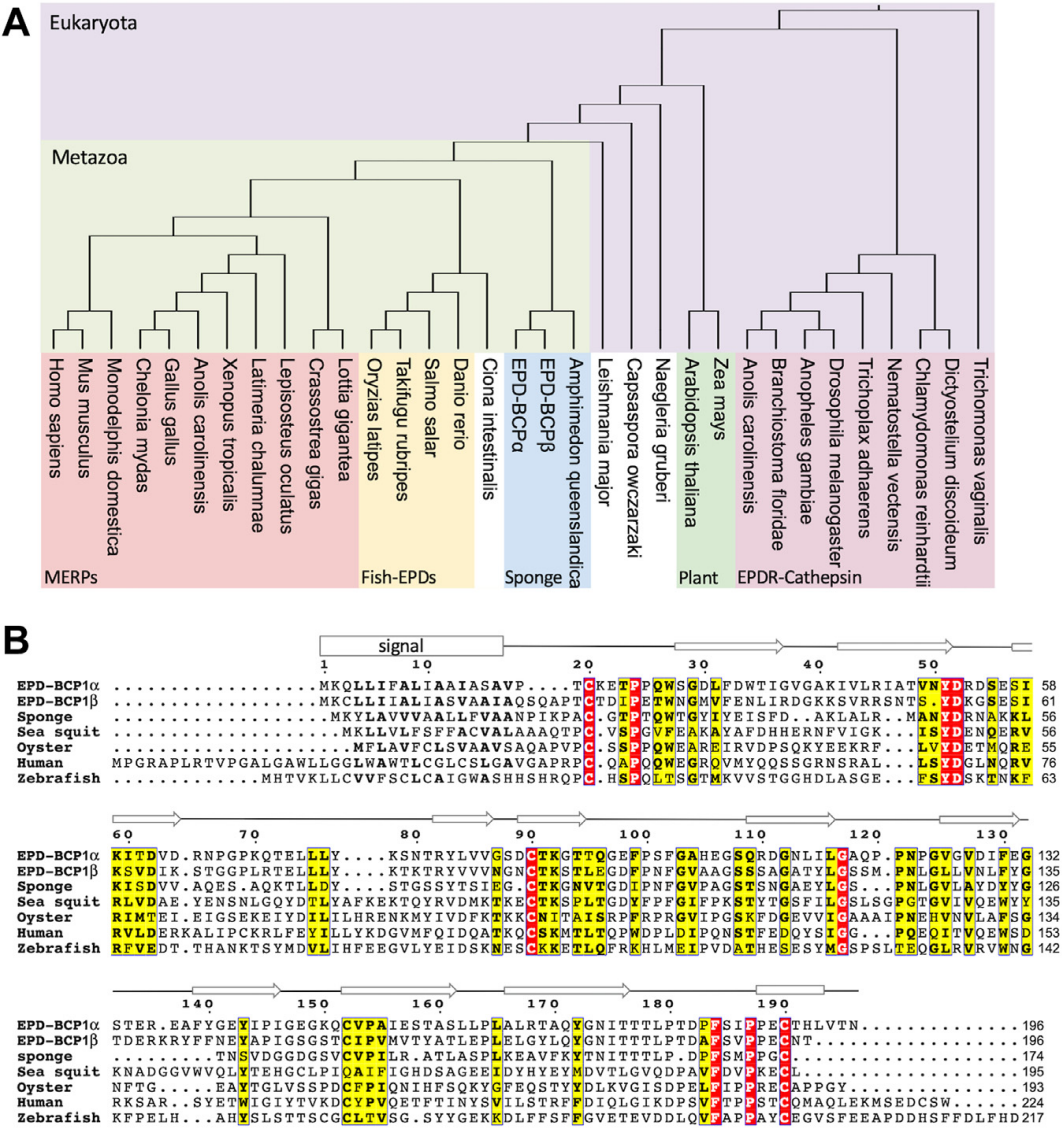
**Phylogenetic analysis of EPD-BCP1.***A*, phylogenetic tree of representative eukaryotic EPDRs including EPD-BCP1 according to the classification in a previous study (28). The accession numbers of protein sequence are listed in Table S4. Species are colored to indicate EPDR1/EPDR1-like MERP group (*red*), fish-specific group (*orange*), sponge group (*blue*), EPDR + cathepsin group (*purple*), and land plant–specific group (*pale green*). *B*, multiple sequence alignment of representative EPDRs of Metazoan lineage. Human MERP (*Homo sapiens*, NP060019), sea squirt EPD (*Ciona intestinalis*, XP_002128930), oyster EPD (*Crassostrea virginica*, XP_022329571), zebrafish EPD (*Danio rerio*, P17561), sponge EPD (*Amphimedon queenslandica*, XP_019856676), EPD-BCP1α (*Haliclona* sp. Kz-2018, LC494533), and EPD-BCP1β (*Haliclona* sp. Kz-2018, LC737963). Conserved signatures are indicated in *yellow boxes*, and invariant residues are indicated in *red*. Residues are numbered according to the EPD-BCP1 sequence, and β-strands and α-helix are indicated by *open arrows* and a *box*, respectively. BCP1, blue carotenoprotein-1; EPD, ependymin; EPDR, EPD-related protein; MERP, mammalian EPD-related protein.

### Crystal structure of EPD-BCP1

The crystal structure of the natively purified EPD-BCP1 was determined by molecular replacement with the AlphaFold2 model (42) and refined to 2.44 Å resolution (Fig. 3*A*). The asymmetric unit contains four dimers, of which the one with the lowest average *B*-factor is described here because the structures of all the dimers are nearly identical. The electron density map delineates N-linked glycosylation on Asn174(α), Asn51(β), and Asn178(β), where two *N*-acetyl-d-glucosamine molecules (*N*-acetyl-β-d-glucosaminyl-(1→4)-*N*-acetyl-β-d-glucosamine) were modeled on each asparagine residue. As predicted from the amino acid sequence (Fig. 2*A*), it seems that Asn92(β) is also glycosylated, but N-glycan was not modeled because of the ambiguity of the electron density in this region. While three pairs of disulfide bonds were found in the crystal structures of EPDR1 (28, 29), EPD-BCP1 lacks two of these cysteine residues in the loop between strands β3 and β4, and at C terminus, resulting in the conservation of two pairs of cysteine residues in each subunit involved in intramolecular disulfide bond formation. The structures of the α and β subunits are very similar with a root mean square deviation of 1.5 Å for 168 Cα atoms, sharing a curved antiparallel β-sheet composed of 11 β-strands (β6-1 and β11-7). Structure comparison of the dimers between EPD-BCP1 and EPDR1 (Protein Data Bank ID: 6E8N) revealed that the two proteins share identical topology of the β-sheet with a root mean square deviation of 3.8 Å for 320 Cα atoms. The outward concave surface of the dimer is formed by all the β-strands except for β7, whereas the inner surface includes the dimer interface composed of eight β-strands (β3-1 and β11-7), which form a tunnel together with the counterpart subunit.

The electron densities corresponding to the two carotenoids are found at the interface between the two subunits. The two carotenoids were identified as AXT and MXT based on the electron density map (Fig. S4). Like in β-crustacyanin (β-CR), the AXT bound to EPD-BCP1 is in the 6/6′-*s*-*trans* conformation (4) in contrast to free AXT, which is mostly in a 6/6′-*s*-*cis* conformation (43). In addition, the β- and κ-end rings of the bound MXT are rotated by around −35° and 170°, respectively, compared with the geometry-optimized NMR structure of free MXT using density functional theory (DFT) calculations at B3LYP/def2-TZVP (Fig. S5). Both the β- and β′-end rings of AXT in EPD-BCP1 are coplanar with the polyene chain as observed in AXTs in β-CR, resulting in the extension of the conjugation system of polyene. In contrast, the two end rings in MXT are noncoplanar with the polyene chain. This should have little effect on the extension of the conjugation system because the κ-end ring has no double bond, and the double bond in the β-end ring is the second adjacent to the C7–C8 triple bond. In EPD-BCP1, AXT and MXT are aligned in a way that one of the pseudo-twofold axes shown in Figure 3*C* is perpendicular to that of the heterodimer, whereas two of the pseudo-twofold axes in β-CR are coincident (Fig. 3*B*). As a common feature of the orientation of the two carotenoids in EPD-BCP1 and β-CR, two polyene planes are nearly parallel with a minimum distance of ∼7 Å, although the carotenoids are slightly bowed outward in EPD-BCP1 but inward in β-CR (Fig. 3*C*). The intersection of the two carotenoids in EPD-BCP1 is around the C14–C15 bond with the C9(MXT)–C15(MXT)–C15(AXT)–C9(AXT) dihedral angle of −141°, whereas that in β-CR is more off-centered around the C12′–C13′ bond with the C6′(AXT1)–C12′(AXT1)–C12′(AXT2)–C6′(AXT2) dihedral angle of 127° (Fig. S6).

### Interaction between protein and carotenoid molecules

The α and β subunits contribute almost equally to the interaction with each carotenoid molecule (Fig. 4, *A*–*C*). Namely, the α-subunit–AXT and β-subunit–AXT interfaces share 48.1% and 45.4% of the total solvent-accessible surface area of AXT, respectively. Similarly, both α-subunit–MXT and β-subunit–MXT interfaces share 46.4% of the total solvent-accessible surface area of MXT. The two carotenoids are accommodated by symmetrically aligned amino acid residues from the two subunits, although the equivalent residue pairs are poorly conserved.

Notably, nine residues occupy the interspace between AXT and MXT in EPD-BCP1 (Fig. 4*A*), in contrast to β-CR, where no amino acid residues were found between the bound AXTs, but a hydrocarbon molecule derived from the paraffin oil used in crystallization was found instead (4). The nine residues in the interface are Leu44(α), Trp34(α), Thr169(α), Ser158(α), and Tyr140(α), and the equivalent residues in the β subunit are Val47(β), Asn38(β), Tyr173(β), and Thr162(β) (but not Phe144(β)). The side chains of Trp34(α), Tyr162(β), Thr169(α), Ser158(α), Tyr140(α), and Asn38(β) form a hydrogen-bond network together with the C8′ hydroxy group in the polyene chain of MXT, which is likely to contribute to the specific interaction between MXT and the binding site, and the polarization of MXT. In addition, the C3′ hydroxy group in the κ-end ring of MXT forms a hydrogen bond with the hydroxy group of Ser133(α), whereas the β-end ring of MXT is fixed by the aromatic ring of Tyr142(β) with a parallel orientation to the C5–C6 double bond (Fig. 4*C*). On the other hand, the C3 hydroxy group and the C4 keto group in the β-end ring of AXT show hydrogen bond interactions with Glu131(α) and Arg49(β)/Lys67(β), respectively, whereas the β′-end ring of AXT appears to fluctuate more because of its weaker interaction with the protein (Fig. 4*B*), as revealed by the higher average *B*-factor of the β′-end ring (56 Å^2^) compared with that of the β-end ring (31 Å^2^).

Apart from the aforementioned residues, the residues within 4 Å of AXT are Asp111(α), Ile129(α), Glu142(α), Ile156(α), Gln171(α), Phe32(α), Ile46(α), Asp64(α), Ala114(β), Tyr134(β), Phe144(β), Glu146(β), Met160(β), Gln175(β), and Phe36(β). Of these residues, Glu142(α) and Glu146(β) are assumed to be protonated to retain the double-hydrogen bonding pairs rather than amide-imido tautomerization of Gln171(α) and Gln175(β) because the existence of negatively charged residues would be unfavorable in the hydrophobic environment. In contrast, the residues within 4 Å of MXT are Ala138(α), Ala160(α), Leu167(α), Ile36(α), Ile42(α), Asn66(α), Tyr142(β), Ala164(β), Leu171(β), Ile40(β), and Lys45(β), which contribute to a hydrophobic environment for the polyene chain of MXT. In contrast to β-CR, no fixed water molecule was found around carotenoids within a distance of 4 Å in EPD-BCP1.

### Reconstitution assays of apoproteins with orange carotenoids

The carotenoid-free apoprotein was obtained by gently mixing the protein solution with diethyl ether and acetone (44). The colorless apoprotein separated into the aqueous phase, and detached orange carotenoids separated into the organic phase. When the apoprotein was reconstituted with the detached orange carotenoids, the solution turned blue and demonstrated an absorption spectrum almost identical to that of the purified holoprotein (Fig. 5*A*).

Next, the apoprotein was tested for its selective binding of carotenoids (44). The apoprotein fully recovered the blue color by reconstitution with AXT and MXT, but the color recovery was incomplete with AXT or MXT alone (Fig. 5*B*). When EPD-BCP1 was reconstituted with fucoxanthin (a marine carotenoid) and canthaxanthin (with a similar structure to that of AXT), each reconstituted protein showed a broad absorbance spectrum with a single peak. The peak did not show a spectral shift, but the shoulder of the peak corresponded to a red shift in each protein (Fig. 5, *C* and *D*). These results indicate that EPD-BCP1 preferentially binds AXT and MXT, and the complete red shift is achieved by the binding of both AXT and MXT.

### Structural insight into the bathochromic shift of carotenoids

Previous theoretical studies of β-CR by quantum chemical calculations (45, 46, 47, 48, 49, 50, 51, 52) have proposed the following three mechanisms underlying the large red shift in the absorption maximum wavelength (*λ*_max_) of ∼100 nm: (1) influences of the conformational changes in AXT upon binding to the protein; (2) polarization effects of the protein environment; and (3) effects of excitonic interactions between the two AXTs. While all three mechanisms are likely involved in the red shift, the extent of their contributions is still controversial. To examine the first mechanism of the red shifts of AXT and MXT upon binding to EPD-BCP1, the effects of the end-ring rotations on the excitation energy were investigated. Geometry optimizations of free AXT and MXT in acetone using DFT calculations at B3LYP/def2-TZVP and subsequent calculations of the vertical S_0_→S_2_ excitation energies at TD-ωB97X/def2-TZVP gave *λ*_max_ values of 469 and 457 nm, respectively (Table 1), comparable to the experimental values of 478 and 474 nm (Fig. 2*B*). In contrast, the geometry optimizations and the excitation energy calculations of AXT and MXT under identical conditions, except for application of dihedral angle constraints to fix the rotations of the two end rings in the protein-bound state, resulted in *λ*_max_ values of 494 and 455 nm, respectively. The C5/C5′–C6/C6′–C7/C7′–C8C8′ dihedral angles of the geometry-optimized free and the protein-bound AXTs were −39.9°/−40.0° and 170.6°/164.4°, respectively, indicating that the former is close to a 6/6′-*s*-*cis* conformation, and the latter is in a 6/6′-*s*-*trans* conformation and nearly coplanar with the polyene chain, as observed in the crystal structure of β-CR. The calculated red shift of 25 nm (0.13 eV) caused by the conformational change from 6/6′-*s*-*cis* to 6/6′-*s*-*trans* is comparable to the values reported in previous theoretical studies on β-CR (45, 46, 51). The calculated energy cost for fixing the torsion angles of the β- and β′-end rings of AXT in the protein-bound state was 1.35 kcal/mol, by which the conjugation system of the polyene chain is extended. In contrast to AXT, there was no red shift in the calculated excited energies between free and protein-bound MXT. This confirms that no extension of the polyene conjugation system occurs in MXT, despite the calculated energy cost of 1.41 kcal/mol for fixing the torsion angles of its β- and κ-end rings in the protein-bound state.

**Table 1:** DFT calculations of vertical excitation energies

Model	*λ*_max_/nm	*f*_osc_
AXT no constrain (6/6′-*s*-*cis*), CPCM (acetone)	469	2.64
MXT no constrain (6/6′-*s*-*cis*), CPCM (acetone)	457	2.71
AXT^a^ ring rotation fixed (6/6′-*s*-*trans*), CPCM (acetone)	494	2.51
MXT^a^ ring rotation fixed (6/6′-*s*-*trans*), CPCM (acetone)	455	2.73
AXT, QM1/QM2/MM	567	2.19
MXT, QM1/QM2/MM	513	2.42
MXT + H_2_O^b^, QM1/QM2/MM	523	2.37
MXTH^+^^c^, QM1/QM2/MM	684	1.81

a The end-ring rotation angles were fixed to those of the protein-bound forms.

b Four water molecules were added in the QM region to connect the amino group of Lys45(β) and the C6′ keto group of MXT *via* a hydrogen-bond network.

c A proton was transferred from N_ζ_ of Lys45(β) to the C6′ keto group of MXT.

Considering the fact that AXT and MXT alone showed red shifts of 71 nm (478→549 nm) and 76 nm (474→550 nm), respectively, upon binding to EPD-BCP1 (Figs. 2*B* and 5*B*), we concluded that the intrinsic effect alone (*i.e.*, conformational changes of the carotenoid itself) could not explain the observed large shifts of *λ*_max_. To evaluate the polarization effects of the protein environment (the aforementioned second mechanism of the red shift), three-layer ONIOM geometry optimizations at the B3LYP-D4/TZVP/HF-3c level were separately performed for each carotenoid. In these optimizations, the QM1 region was defined as each carotenoid, the QM2 region as the side chains of the nearby hydrophilic and aromatic residues, and the MM region as the rest of the whole protein including N-glycans, as well as the solvent molecules and ions. Subsequent QM calculations of the excitation energies of AXT and MXT at the TD-ωB97X/def2-TZVP level for the geometry-optimized QM1 and QM2 regions resulted in *λ*_max_ values of 567 and 513 nm, respectively (Table 1), implying that the polarization effects of the protein environment also could not fully explain the large red shift of MXT upon binding to EPD-BCP1. To evaluate the effects of the protein environment on the geometry-optimized ground state structures of AXT and MXT in the protein, we calculated the bond length alternation (BLA) values. The BLA is defined as the average difference between single- and double-bond lengths in a π-conjugated system, and a correlation between the BLA values and the excitation energies has previously been reported (52). The BLA values of the geometry-optimized structures of AXT in acetone and EPD-BCP1 were 0.075 Å and 0.045 Å, respectively, whereas those of the geometry-optimized structures of MXT in acetone and the protein were 0.066 Å and 0.057 Å, respectively. The larger difference in the BLA values of AXT between those in acetone and the protein compared with those of MXT is consistent with the larger calculated red shift of AXT upon binding to the protein. Furthermore, it is interesting to note that the β-end ring side of AXT, which shows more intimate contact with the protein, has a much smaller BLA compared with the β′-end ring side (Fig. S7). The same tendency was found for the κ- and β-end ring sides in MXT, although the difference was more modest than that in AXT.

It is most likely that the direct interaction between AXT and the two positively charged residues, Arg49(β) and Lys67(β), plays a key role in the red shift, although their charges should be neutralized by the two adjacent acidic residues, Asp111(α) and Glu131(α) (Fig. 4*B*). In the case of MXT, the Nζ atom of Lys45(β) is at a distance of 5.6 Å from the C6′ carbonyl oxygen, which is not neutralized by any acidic residues. We first assessed the effect of the indirect interaction between Lys45(β) and MXT *via* a hydrogen-bond network involving four water molecules, which were identified in the QM1/QM2/MM geometry–optimized structure but not in the electron density map. The QM calculation of the excitation energy with the four water molecules included in the QM region gave a *λ*_max_ of 523 nm (Table 1), which was closer to the experimental value of 550 nm. We further assessed the possibility that a proton of Nζ of Lys45(β) is transferred to the C6′ keto group of MXT because it has been known that protonation of the C4 keto group of AXT causes a significant red shift (46). The geometry optimization caused a larger displacement of the nearby residues, and subsequent QM calculation of the excitation energy resulted in a *λ*_max_ of 684 nm, which was rather overestimated compared with the experimental value.

Assuming that each carotenoid binds specifically to their own binding site in EPD-BCP1, it is speculated that difference in *λ*_max_ between the double- and single-carotenoid–bound EPD-BCP1 would be mainly attributable to the exciton coupling effect. The experimental values of *Δλ*_max_ between the double- and single-carotenoid–bound EPD-BCP1 were 7 to 8 nm (Fig. 5*B*), confirming that the effect of exciton interaction of the two carotenoids on the large red shift is marginal compared with that of the polarization effect by the protein environment as previously reported in the theoretical studies on β-CR (48, 49).

### Phylogeny and diversity of EPD-BCP1

Although true EPDs are restricted to teleosts, the distribution of the EPDR protein family is much broader than originally thought. McDougall *et al.* (23) examined the phylogeny of EPDRs and reported considerable diversity among bilaterian animals and other eukaryotes. They classified EPDRs on the basis of the pattern of conserved cysteine residues in profiles 1 to 3, which supported the phylogenetic division of EPDRs into "clade 1" and "clade 2". Accordingly, the sponge EPDs encoded in the genome of *A. queenslandica* were placed in an ancestral clade within clade 1, which included the clades of mammalian ependymin-related proteins and original fish-EPDs (23). A more recent classification based on protein structure indicated that EPDRs belong to the LolA (bacterial proteins related to the LolA lipoprotein transporter)/EPDR superfamily (28). Phylogenetic analysis revealed that EPD-BCP1 constitutes a sponge clade with *A. queenslandica* EPDR in an early branching metazoan lineage (28) (Fig. 6*A*) and displays profile-1 in the previously described "clade 1" subgroup based on the conserved cysteine residue patterns (23) (Fig. 6*B*). To our knowledge, none of these EPDs are identified as color proteins.

## Discussion

In this study, a water-soluble BCP was purified from the marine sponge *Haliclona* sp. Several marine animals harbor orange carotenoids, but they display blue color (8). However, the molecular mechanisms underlying coloration in these animals remain largely unknown. To the best of our knowledge, EPD-BCP1 is the second BCP to be characterized structurally and functionally in marine organisms. Hence, this carotenoprotein may be useful to study the mechanisms underlying the bathochromic shift.

EPD-BCP1 is a member of the EPDR family, whose members have diverse functions. They were originally identified as fish-specific secreted glycoproteins (21, 22). Although the functions of protein homologs in the annotated genome data of other species remain largely uncharacterized, previous studies have suggested their involvement in the fish nervous system, intestinal regeneration in sea cucumbers (53), binding of the calcium-dependent matrix because of the presence of N-linked carbohydrate moieties (54), human fibroblast contractility (55), and the development of human brown fat cells (27). Recently, Wei *et al.* (28) and Park *et al.* (29) analyzed the X-ray structures of EPDR1 and identified the relationship between the EPDR family and the bacterial LolA lipoprotein family based on the presence of an LolA fold in these proteins. On the basis of its protein structure and *in vitro* lipid-binding profile, EPDR1 is believed to be involved in lipid binding and transport (28, 29). Although the functional role of EPDR1 remains unclear because of its yet-uncharacterized natural binding of lipids, the results of this study provide convincing evidence of the ability of sponge EPDR to bind lipophilic carotenoids. Nevertheless, further studies are required to elucidate the evolution and function of these EPDs, not only to unravel their possible involvement in the coloration of marine organisms but also to reveal the roles of functionally uncharacterized EPDRs in vertebrates and invertebrates. While there is no direct experimental data regarding the ligand-binding site of EPDR1, the previous crystal structure analysis of EPDR1 from human in a PEG-bound form suggested that the ligands bind to the outer concave surface of the β-sheet (28). In contrast, the crystal structure of the native EPD-BCP1 in this study revealed that the two carotenoids bind to the dimer interface in a specific manner, leaving the deep outer clefts of the β-sheets vacant. We note, however, that PEG was used as a precipitant for crystallization, and the concave surface of EPD-BCP1 is rich in hydrophobic residues, like in EPDR1. On the other hand, the entrance into the dimer interface of EPDR1 is blocked mainly by the loop between the β3 and β4 strands, and the surface of the dimer interface is rich in hydrophilic residues compared with that of EPD-BCP1. These findings suggest that the dimer interface of EPDR1 is unsuitable for the binding of the hydrophobic ligands.

Here, the two carotenoids were identified from spectroscopic data as (3*S*,3′*S*)-AXT (peak P1) and MXT (peak P2). In crustaceans, AXT is a mixture of (3*S*,3′*S*)-, (3*R*,3′*S*)-, and (3*R*,3′*R*)-isomers, which may be produced from the ingested food. In contrast, blue sponge contains only (3*S*,3′*S*)-AXT. This may be because it is produced from the usual (3*R*,3′*R*)-zeaxanthin by ketolase or from β-carotene by a stereospecific hydroxylase and ketolase, which could be included in blue sponge or in unknown symbiotic bacteria. Notably, the hydroxyl groups of (3*S*,3′*S*)-AXT and (3*R*,3′*R*)-zeaxanthin share the same chirality. MXT, which was first isolated from the edible mussel *Mytilus edulis*, is widely distributed among marine animals (37, 38) but not among carotenoid-producing organisms. MXT is produced from the fucoxanthin of brown algae (38). Further studies are warranted to clarify the enzymes and metabolic pathways associated with these carotenoids.

Our X-ray crystal structure analysis of EPD-BCP1 has revealed some striking similarities with β-CR (4) in terms of the structural properties of the bound carotenoids, although these two carotenoproteins are evolutionarily unrelated. Both proteins form heterodimers that bind two carotenoid molecules in a way that two polyene planes are nearly parallel with a minimum distance of ∼7 Å. In addition, the end-ring rotation angles of AXT in EPD-BCP1 are very close to those found in β-CR, resulting in the 6/6′-*s*-*trans* conformations of AXT. The DFT calculations in this study have confirmed that the end-ring rotations alone partially contribute to the red shift of AXT upon binding to EPD-BCP1 (469→494 nm according to the DFT calculations *versus* experimental values of 478→549 nm). These findings are in good agreement with those of previous theoretical studies on β-CR (45, 46, 51). In contrast, the end-ring rotations of MXT upon binding to EPD-BCP1 make almost no contribution to the red shift (457→455 nm according to the DFT calculations), regardless of the large rotations of the two end rings, as mentioned before. The influences of the protein environment on the red shifts of AXT and MXT were estimated on the basis of QM1/QM2/MM geometry optimizations and subsequent excitation energy calculations. Consequently, the calculated *λ*_max_ of 567 nm for AXT in the protein is comparable to the experimental value of 549 nm, whereas the calculated *λ*_max_ of 513 nm for MXT is much shorter than the experimental value of 550 nm. Further studies with spectroscopic and theoretical approaches will elucidate the mechanism of the bathochromic shift of MXT upon binding to EPC-BCP1.

Previously, an AXT-binding carotenoprotein in a photo-oxidative stress–tolerant eukaryotic microalga was identified as the first water-soluble carotenoprotein in eukaryotic plants with an N-terminal signal sequence for cell-surface secretion. EPD-BCP1 is also a water-soluble AXT- and MXT-binding carotenoprotein expressed on the cell surface. Considering the roles of AXT and MXT in providing protection from sunlight and in scavenging singlet oxygen, these proteins may be primarily adapted to localize the carotenoids on the cell surface. Because carotenoid-binding proteins vary widely and have evolved independently across taxonomic groups, further research is required to clarify the diversity, evolution, and functional commonality of the selective binding of different carotenoid species among a wide range of organisms.

## Experimental procedures

### Animal material and sponge characterization

Blue sponges were collected from the coral reef off the coast of Okinawa prefecture, with the help of a fish company between 2016 and 2021. Collecting marine blue sponges is not prohibited in Japan. Our study did not use laboratory animals and not needed ethics oversight. Type specimens were deposited into the collection of the National Museum of Nature and Science as a deposit number NSMT-Po-2491 (NSMT). The sponge samples were classified by morphological and genotypic characterization with an expert sponge taxonomist. Briefly, fresh sponge samples were fixed in 95% ethanol solution for morphological observation of spicule type, skeletal formations, and architectural structures. The skeletal structure and scleral features were observed under an optical microscope. The morphological features of the sponge samples were matched to those of the genus *Haliclona* as described in “Systema Porifera: A Guide to the Classification of Sponges” (30, 31). Genomic DNA was extracted from sponge tissues using a DNeasy Tissue Kit (Qiagen) in accordance with the manufacturer’s protocol. The Cox1 gene was amplified by PCR using the primers FCo1490 (5′-GGTCAACAAATCATAAAGAYATYGG) and RCo2198 (5′-TAAACTTCAGGGTGACCAAARAAYCA) (32, 33). The resulting PCR products were purified using the QIAquick PCR purification kit (Qiagen) and sequenced by Macrogen. The sequences of the *cox1* genes (658 bp) obtained from sponge samples collected in 2016 and 2018 were identical and used to search for sequence similarities. These sequences and those of taxonomically related species were aligned using the ClustalW for phylogenetic analyses (56). Phylogenetic trees were constructed using the maximum-likelihood methods with MEGA X (57).

### Purification procedures and determination of peptide sequences

Fresh sponge samples were gently squeezed, and the released blue droplets were collected as crude extracts. About 1 ml of blue extract (Abs_557_ = 2.0) was obtained from 1.0 g of sponge tissue after removing excess seawater. Aqueous supernatants were obtained after ultracentrifugation at 100,000*g* for 2 h. A single aqueous blue fraction was collected by passing through a gel-filtration column (HR100; GE Healthcare) and DEAE Sepharose fast flow column (GE Healthcare) using 50 mM Tris–HCl buffer, pH 7.0. Final purification yields of the blue protein were 50 to 70%. After the blue fraction was concentrated, the purified proteins were separated by SDS-PAGE. The molecular weights of the proteins were estimated using a commercial marker kit (Precision Plus Protein; Bio-Rad). The N-terminal amino acid sequence was determined by the Edman degradation method using a peptide sequencer (PPSQ30; Shimadzu). The formyl groups were deblocked by soaking the transferred membrane overnight in 100 mM HCl solution. Periodic acid–Schiff staining was performed with a commercial staining kit (Merck) in accordance with the manufacturer’s instructions. The molecular masses of the purified proteins were determined by gel-filtration chromatography and calibrated with the following molecular standards (Pharmacia): ribonuclease (13.7 kDa), carbonic anhydrase (29 kDa), ovalbumin (44 kDa), conalbumin (75 kDa), aldolase (158 kDa), thyroglobulin (670 kDa), and blue dextran (2000 kDa).

### Carotenoid extraction and identification

Carotenoids were extracted from the purified carotenoprotein by the Bligh–Dyer method1 as successive extractions with methanol:chloroform (5:2) by gentle mixing in a tube. After the addition of chloroform and then water (2:3), the organic phase was obtained and evaporated to dryness under nitrogen gas. The extracted carotenoids were completely dissolved in acetone.

Carotenoids were identified using an HPLC photodiode array system (200–700 nm) (L-2455; Hitachi) equipped with a Capcel PAK C18 reversed-phase column (150 × 4.6 mm i.d., 5 μm particle size; Shiseido). The solvent system was a mixture of methanol/water (80/20, v/v, solvent A) and a mixture of acetone/methanol (1/1, v/v, solvent B). The column was eluted at a flow rate of 1.0 ml min^−1^ with a linear gradient: 0 to 4 min, 75% A:25% B to 50% A:50% B; 4 to 20 min, 50% A:50% B to 100% B; and 20 to 50 min, 100% B.

The same column system described previously was used for LC–MS. Positive ion mass spectra were recorded in full scan mode (*m/z* 70–1000) with an electrospray ionization source and a Q-Exactive Focus Orbitrap LC–MS/MS System (ThermoFisher Scientific). The interface voltage was set at 4.5 or −3.5 kV. Nitrogen gas was used as nebulizing gas at a flow rate of 1.5 l min^−1^. Charged droplets and heat block temperatures were both 200 °C. The data of molecular masses in high-resolution LC–MS were analyzed with Compound Discoverer, version 2.1 software (ThermoFisher Scientific).

^1^H-NMR (500 MHz) spectra of the peak P1 and peak P2 components of CDCl_3_ were measured using a Unity Inova-500 system (Varian). The chirality of AXT was determined using a Sumichiral OA-2000 column (Agilent Technologies) with *n*-hexane/chloroform/ethanol (48:16:1 by volume) as the eluent at a flow rate of 1.0 ml/min (58). (3*R*,3′*R*)-AXT from red yeast *Xanthophyllomyces* was used as a control.

### Crystallization, X-ray diffraction data collection, and structure determination

The natively purified EPD-BCP1 was crystallized by the sitting drop vapor diffusion method at 23 °C, where the inner drops were prepared by mixing 1.0 μl of 9.8 mg/ml protein in 10 mM Tris–HCl (pH 7.5) and 1.0 μl of reservoir solution. The best crystal was grown within 4 weeks with the reservoir solution consisting of 0.1 M Tris–HCl (pH 8.5), 0.2 M MgCl_2_, and 20% (w/v) PEG 8000. The crystal was transferred to the reservoir solution supplemented with 20% ethylene glycol for cryoprotection and flash-frozen in liquid nitrogen. X-ray diffraction data were collected at the beamline BL-5A of KEK Photon Factory at a wavelength of 1.0000 Å at 100 K. The diffraction data were integrated and scaled with iMosflm and Aimless from CCP4 software suite (59), respectively. The phases were determined by molecular replacement using MOLREP (59), where the AlphaFold2 (42) model calculated from the amino acid sequence of the α subunit of EPD-BCP1 was used as a search model. The subsequent model building and structure refinement were iteratively performed with Coot (60) and Refmac5 (61), respectively. The restraint CIF files for AXT and MXT were prepared based on the QM1/QM2/MM geometry–optimized structure. The data collection and refinement statistics are summarized in Table S3, where the Ramachandran analysis was performed with Rampage (62). Structure figures were prepared with PyMol (The PyMOL Molecular Graphics System, version 2.4.0; Schrödinger, LLC) and Avogadro (63).

### Preparation of apoprotein and its reconstitution with carotenoids

Apo-EPD-BCP1 was obtained by treating the purified protein with organic solvents diethyl ether/acetone (1/1). AXT and MXT were extracted from the purified EPD-BCP1 as described previously. Canthaxanthin and fucoxanthin were extracted from microalgal AstaP-orange1and blown alga *Eisenia* sp., respectively (18, 44). Briefly, the crude extracted pigments were dissolved in hexane and were separated by silica gel HPTLC with concentrate zone (Merck; catalog no.: 1.13748.0001) and developed with dichloromethane–ethyl acetate–acetone (1:2:1, by volume). The extracted carotenoids were further collected by C18-HPLC. The authenticity of the purified carotenoids was determined based on the absorption spectra obtained using an HPLC photodiode array detector, HPLC retention times, and molecular masses from high-resolution LC–MS analysis in comparison with those of standard compound (18, 19, 44). Apo-EPD-BCP1 was used for reconstitution assays after removing the organic solvents under an N_2_ gas stream. Apoprotein (dissolved in 50 mM Tris–HCl buffer [pH 7.5]) and a small amount of carotenoid solutions (dissolved in acetone) were mixed together and incubated overnight on ice (44). Unbound insoluble carotenoids were removed by centrifugation for 5 min at 15,000*g*, and the trace amounts of free carotenoids were removed by brief extraction with diethyl ether followed by removing the residual diethyl ether under an N_2_ gas stream. Absorption spectra of apo- and holo-EPD-BCP1 were measured using a spectrophotometer (Shimadzu UV-1000). A blank experiment (without protein) did not produce detectable carotenoids by this method.

### Quantum chemical calculations

All density functional calculations including three-layer ONIOM geometry optimizations were performed with ORCA (5.0.3) (7, 8). The geometry optimizations of carotenoids in solution were carried out with the B3LYP functional and the def2-TZVP basis set, where the conductor-like polarizable continuum model method (9) has been employed to mimic the experimental environment. The excitation energies were subsequently calculated under the same conditions except that TD-ωB97X was used as the functional basis set. The geometry optimizations of AXT and MXT in EPD-BCP1 were separately conducted with the three-layer ONIOM (QM1/QM2/MM) method, where the B3LYP-D4 functional and the def2-TZVP/HF-3c basis sets were used. The initial model and the parameter files were prepared with AmberTools20 (7, 8) and Amber20 (10). The model included the α and β subunits of EPD-BCP1, two carotenoids, and N-glycans, whose parameters were obtained with ff14SB, GAFF, and GLYCAM_06j-1, respectively. The protonation states of the ionizable residues under a physiological condition were evaluated with H++ (4.0) (11), which has suggested that Glu142(α) and Glu146(β) are protonated as expected. The solvation was performed in the 12-Å OPCBOX with the TIP3P model, where the 50 sodium ions and 44 chloride ions were included. A series of steps for energy minimization were conducted with all nonhydrogen atoms frozen except for water molecules and ions. For the geometry optimization, each carotenoid was assigned as the QM1 region, and the hydrophilic or aromatic residues within the distance of 4 Å from each carotenoid molecule were basically assigned as the QM2 region. Specifically, the QM2 region for AXT consists of the atoms in the side chains of Phe32(α), Trp34(α), Asp111(α), Glu131(α), Tyr140(α), Glu142(α), Ser158(α), Thr169(α), Gln171(α), Phe36(β), Asn38(β), Arg49(β), Lys67(β), Tyr134(β), Phe144(β), Glu146(β), Thr162(β), Tyr173(β), and Gln175(β). On the other hand, the QM2 region for MXT includes the atoms in the side chains of Trp34(α), Asn66(α), Glu131(α), Ser133(α), Tyr140(α), Ser158(α), Thr169(α), Asn38(β), Ly45(β), Lys67(β), Tyr142(β), Phe144(β), Thr162(β), and Tyr173(β). In addition to the atoms in the QM1 and QM2 regions, the atoms in the side chains of aliphatic residues within the distance of 4 Å from each carotenoid molecule were assigned to be flexible during the geometry optimizations. The subsequent calculations of the excitation energies were performed for the atoms in the QM1 and QM2 regions with the TD-ωB97X functional and the def2-TZVP basis set.

### Construction of a cDNA library and cloning of cDNA encoding EPD-BCP1

Total RNA was extracted with Trizol reagent (ThermoFisher Scientific) and reverse transcribed into cDNA, which was used to generate a full-length cDNA library with the SMARTer Pico PCR cDNA Synthesis Kit (Takara Bio) in accordance with the manufacturer’s instructions. The cDNA libraries were sequenced using an Illumina HiSeq 2500 system (Illumina). FASTQ files were imported to the CLC Genomics Workbench (QIAGEN), and *de novo* sequence assembly was performed. Approximately, 30,000 contigs after the *de novo* assembly were generated by the CLC Genomics Workbench. To confirm the nucleotide sequence of cDNAs encoding the N-terminal sequences of the EPD-BCP1α and EPD-BCP1β, the cDNAs were amplified by PCR using the cDNA library as a template. The amplified PCR product was sequenced, and the full-length cDNA sequence was confirmed by juxtaposing sequences of the 5′ and 3′ portions. The N-terminal signal peptide sequences were predicted by the SignalIP-5.0 Server (http://www.cbs.dtu.dk/services/SignalP/).

### Phylogenetic analysis of EPD-BCP1

A phylogenetic tree of EPD-BCP1 was initially aligned using ClustalW and generated by maximum likelihood tree with RAxML using the best model (GTR-GAMMA) (64). The accession numbers of the protein sequences used for phylogenetic analysis are listed in supporting information 1. Alignment figures were prepared with ESPript 3.0 (65). Tree was drawn with Itol, version 6 (https://itol.embl.de/) (66).

## Data availability

Type specimens were deposited into the collection of the NSMT (accession number: NSMT-Po-2491). Accession numbers and source data are presented in the main text and supporting information. All data used to support the findings of this study have been included in the article and supporting information or made available through the National Center for Biotechnology Information. The sponge proteins are available from corresponding author upon reasonable request. The atomic coordinates and structure factors for EPD-BCP1 have been deposited in the Protein Data Bank under the accession number of 8I34.

## Supporting information

This article contains supporting information (39, 67).

## Conflict of interest

The authors declare that they have no conflicts of interest with the contents of this article.
