# Supplementary material for: An ependymin-related blue carotenoprotein decorates marine blue sponge
Source: J Biol Chem. 2023 Jul 28;299(9):105110. doi: 10.1016/j.jbc.2023.105110 (PMC10470211; doi:10.1016/j.jbc.2023.105110)
Supplement: Supporting Figures S1–S7 and Tables S1–S4 [file mmc1.pdf]

Supporting information

An ependymin-related blue carotenoprotein decorates marine blue sponge

Shinji Kawasaki[1,4]*, Takayuki Kaneko[1,4], Tomomi Asano[1], Takashi Maoka[2], Shinichi Takaichi[1], Yasuhito Shomura[3]*

[1]Department of Molecular Microbiology, Tokyo University of Agriculture, 1-1-1 Sakuragaoka, Setagaya-ku, Tokyo 156-8502, Japan
[2]Research Institute for Production Development, 15 Shimogamo, Morimoto Cho, Sakyoku, Kyoto, 606-0805, Japan
[3]Institute of Quantum Beam Science, Graduate School of Science and Engineering, Ibaraki University, 4-12-1 Nakanarusawa, Hitachi, Ibaraki, 316-8511, Japan
[4]Co-first authors
*Correspondence: kawashin@nodai.ac.jp (S.K.), yasuhito.shomura.s@vc.ibaraki.ac.jp (Y.S.)

Supplemental Figures and Tables

Figure S1. Morphology of sponge samples used in this study 2
Figure S2. Maximum likelihood phylogenetic tree based on the *cox1* gene 3
Figure S3. Purification of blue protein from the samples collected in 2016 4
Figure S4. Two carotenoids in EPD-BCP1 5
Figure S5. Theoretical solution structures of carotenoids 6
Figure S6. Comparison of the orientations of the two bound carotenoids in EPD-BCP1 and β-CR 7
Figure S7. Comparison of the carbon-carbon bond lengths of the DFT models of the two carotenoids 8
Table S1. ^{1}H-NMR data of peak-P1 carotenoid and astaxanthin in $CDCl_3$ 9
Table S2. ^{1}H-NMR data of peak-P2 carotenoid and mytiloxanthin in $CDCl_3$ 10
Table S3. Data collection and refinement statistics 11
Table S4. Information of proteins used for phylogenetic analysis in Figure 6A. 12
Supplementary references 13

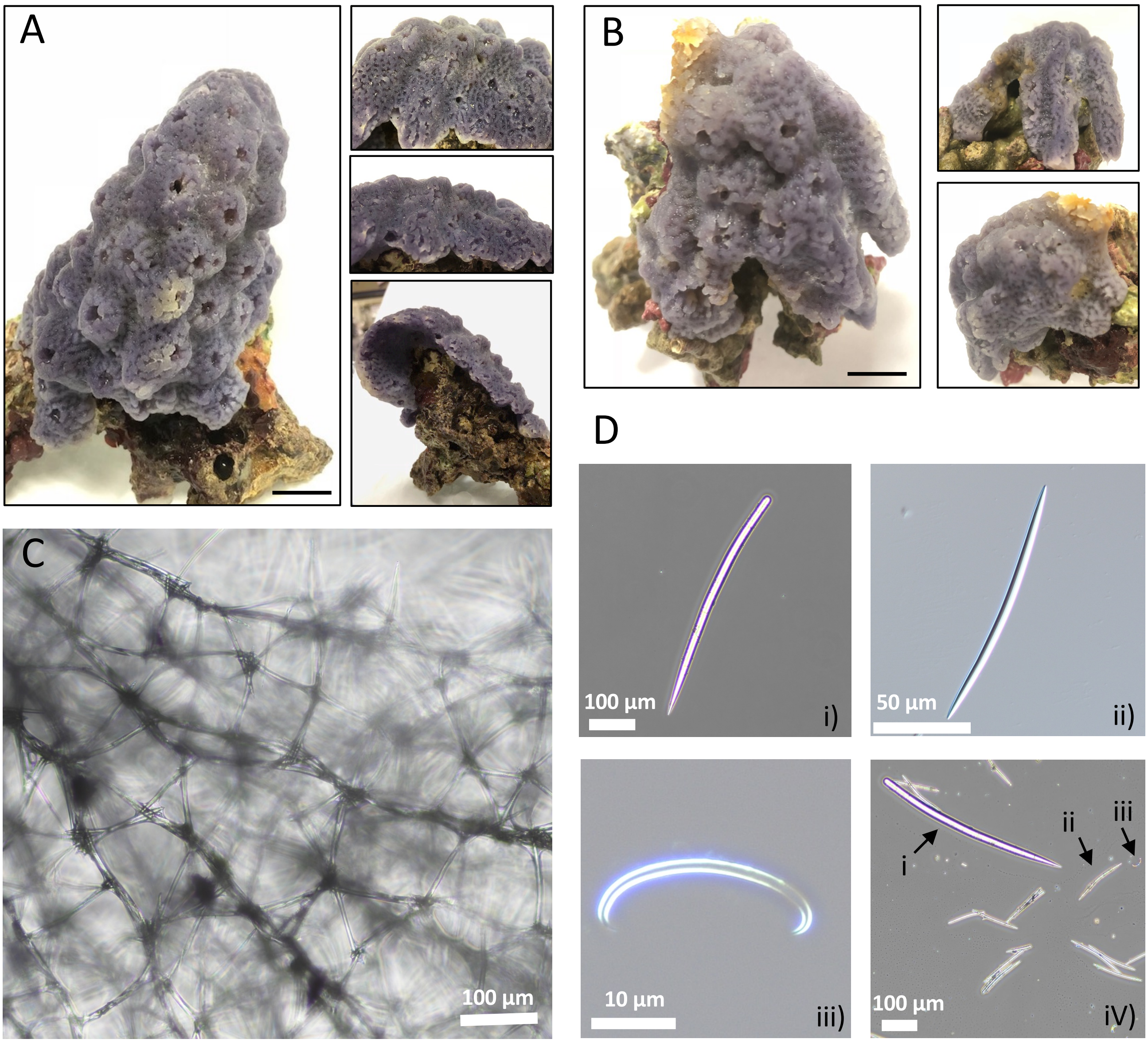

Figure S1. Morphology of sponge samples used in this study. Pictures of the blue sponges collected in 2018 (*A*) and 2016 (*B*). Scale bar = 3.0 cm. *C*, Peripendicular section of choanosomal skeleton. Primary line and secondary line are regularly connected. *D*, Spicule morphotypes. Oxeas are gently curved. i) oxeas type I, ii) oxeas type II. (iii) sigmas are c-shaped. (iv) oxeas of different sizes.

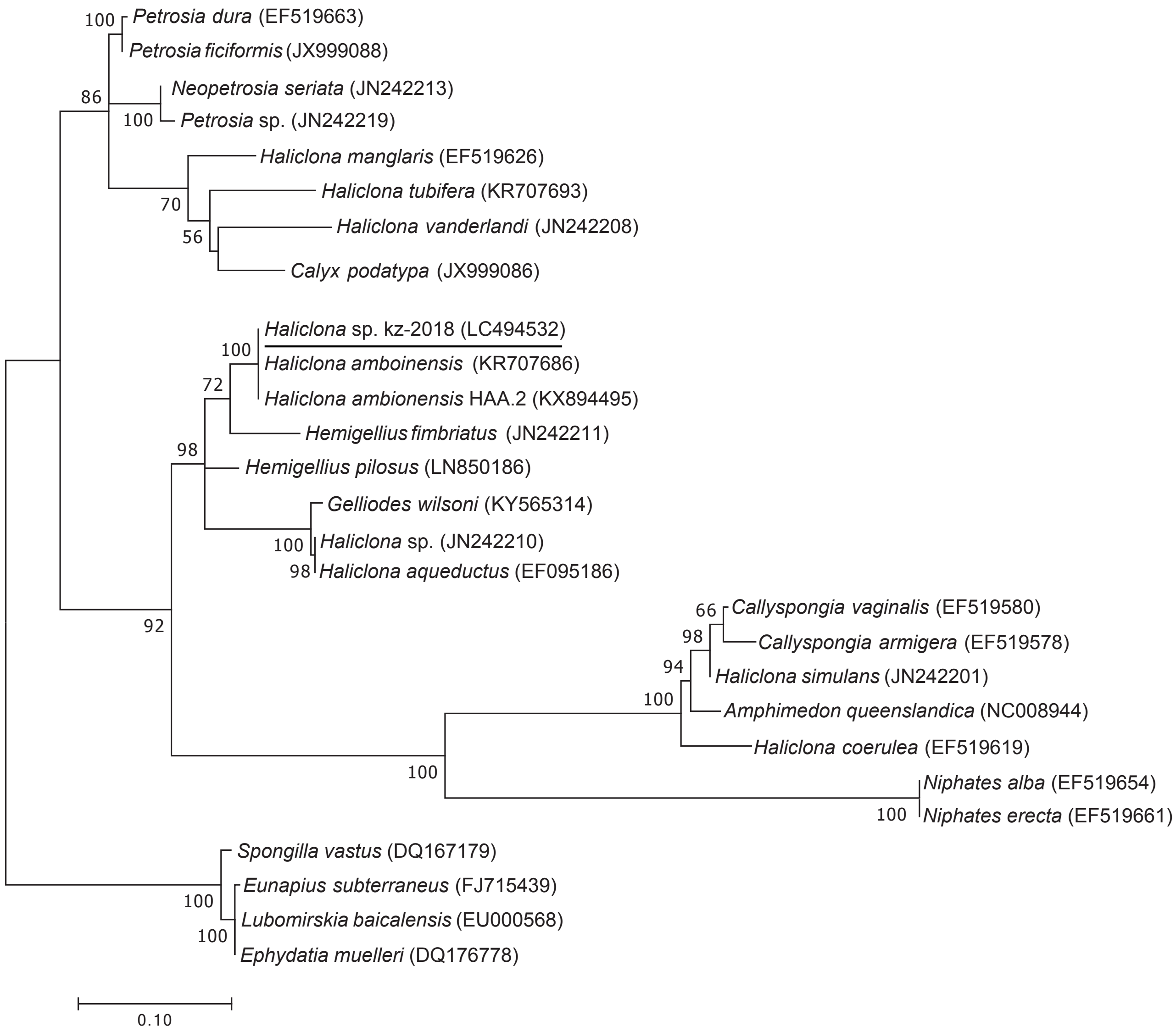

Figure S2. Maximum likelihood phylogenetic tree based on the *cox1* gene. The numbers at nodes indicate the bootstrap values obtained by MEGA-X. The bootstrap proportions (>50%) are shown next to the branches. The tree is rooted in the freshwater haplosclerids *E. muelleri*, *E. subterraneus*, *S. vastus*, and *L. baicalensis.* GenBank accession numbers are indicated.

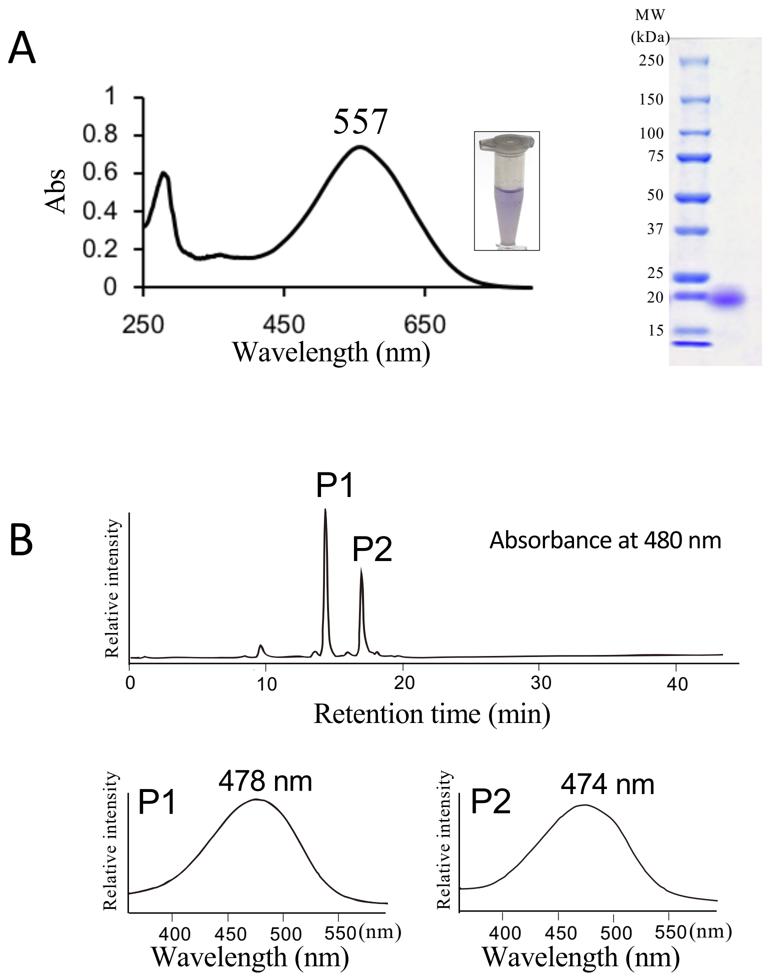

Figure S3. Purification of blue protein from the samples collected in 2016. *A,* Absorption spectrum and SDS-PAGE profile of the blue protein purified from the samples collected in 2016. Color of the purified protein is shown in the inset. *B,* HPLC elution profiles of bound carotenoids using an HPLC photodiode array detector. The spectrum of each peak P1 and P2 is shown in below.

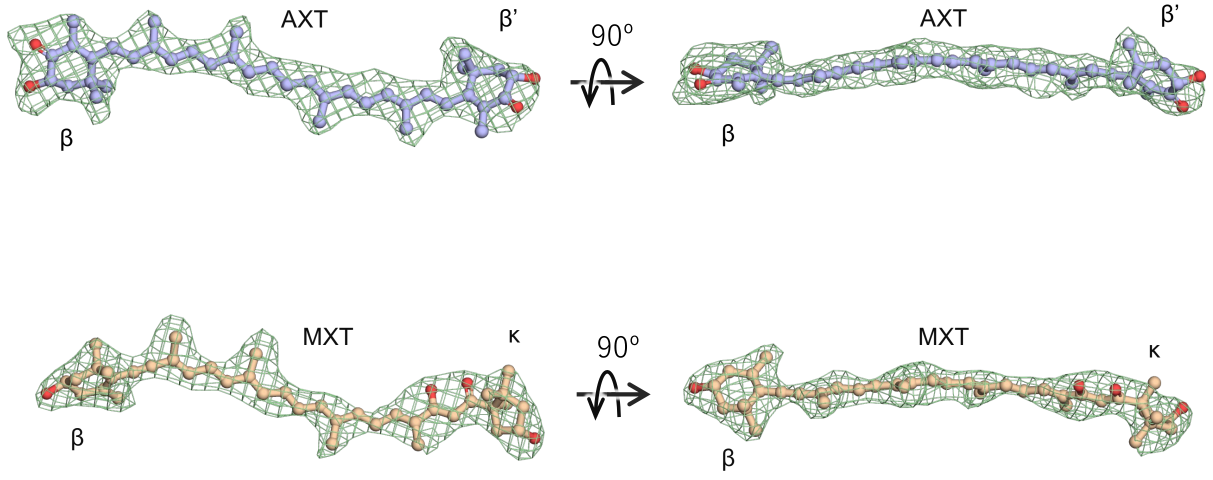

Figure S4. Two carotenoids in EPD-BCP1. Refined models of AXT and MXT and their $F_o - F_c$ omit electron density maps contoured at 2.5 σ.

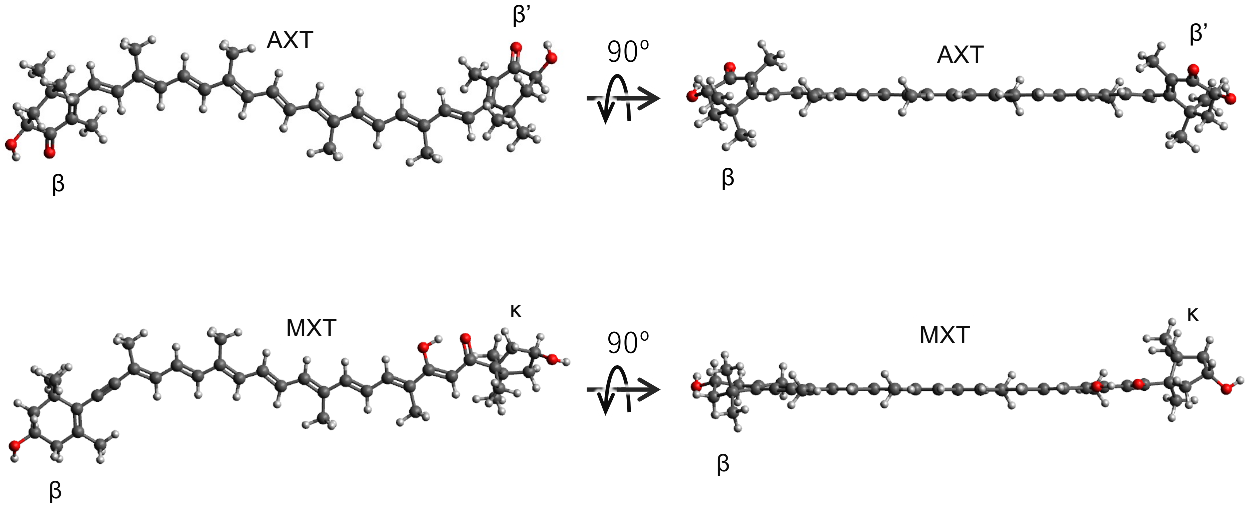

Figure S5. Theoretical solution structures of carotenoids. Geometry-optimized structures of AXT and MXT using DFT calculations in CPCM (acetone) at B3LYP/def2-TZVP level.

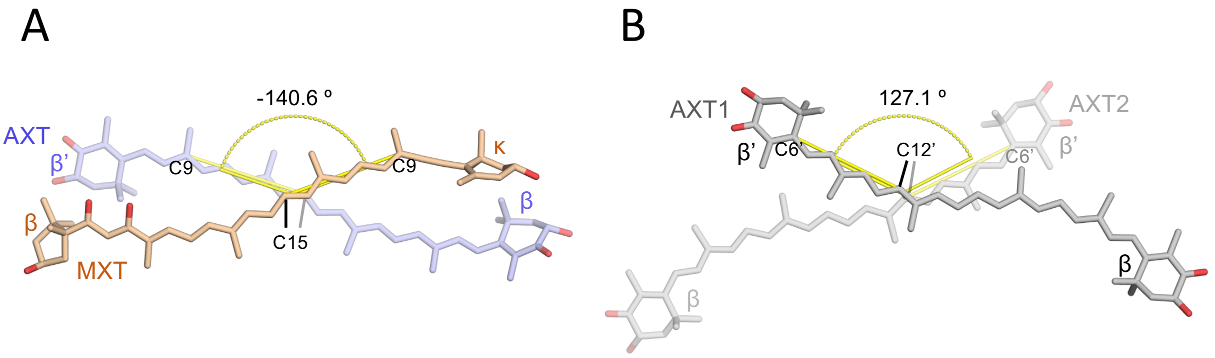

Figure S6. Comparison of the orientations of the two bound carotenoids in EPD-BCP1 and β-CR. *A,* AXT and MXT in EPC-BCP1. *B,* AXT1 and AXT2 in β-CR. Carbon atoms used in the definition of the dihedral angles are labeled.

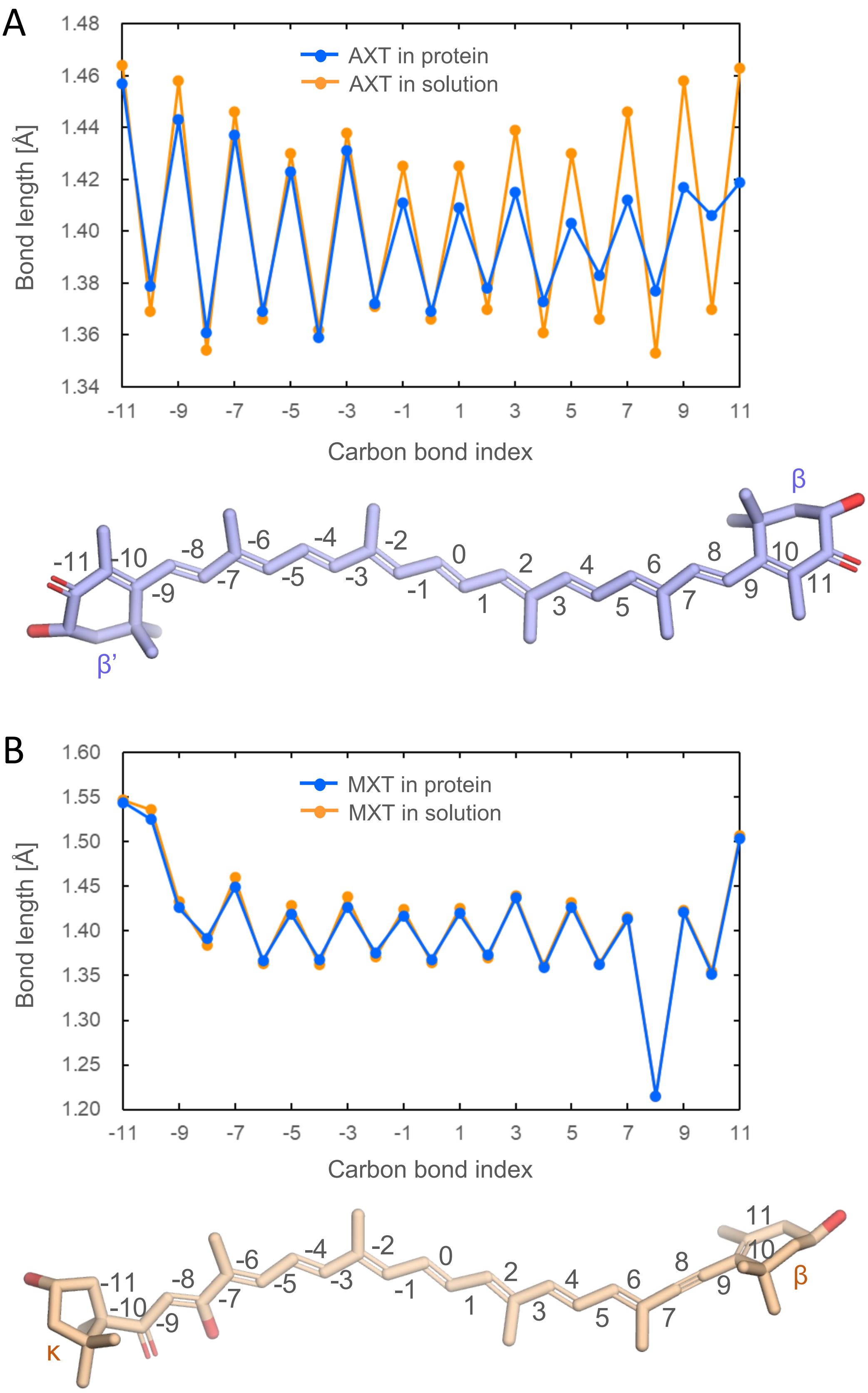

Figure S7. Comparison of the carbon-carbon bond lengths of the DFT models of the two carotenoids. *A,* AXT in protein (QM1/QM2/MM) and in solution (no constrain in acetone). *B,* MXT in protein (QM1/QM2/MM) and in solution (no constrain in acetone).

Table S1. ^{1}H-NMR data of peak-P1 carotenoid and astaxanthin in $CDCl_3$

	d Values in ppm, multiplicity	
H	Peak-P1	Astaxanthin[1]
H-2 ax	2.16 ddd	2.15
H-2 eq	1.82 dd	1.82
H-3	4.33 dd	4.30
HO-3		3.68 d
H-7	6.22 d	6.22
H-8	6.43 d	6.43
H-10	6.30 d	6.30
H-11	6.66 dd	6.66
H-12	6.45 d	6.45
H-14	6.31 d	6.30
H-15	6.67 dd	6.67
CH_3-16	1.326 s	1.323 s
CH_3-17	1.214 s	1.211 s
CH_3-18	1.948 s	1.945 s
CH_3-19	2.004 s	2.001 s
CH_3-20	1.994 s	1.991 s

s, singlet; d, doublet

Table S2 ^{1}H-NMR data of peak-P2 carotenoid and mytiloxanthin in $CDCl_3$

	d Values in ppm, multiplicity	
H	Peak-P2	Mytiloxanthin[2]
H-2	~ 2.18	2.19 dd
H-2	1.72 dd	1.72 dd
H-3	4.53	4.53 dddd
H-4	2.88 dd	2.88 dd
H-4	~ 1.54	1.55 dd
H-7	5.862 s	5.861 s
H-10	7.24 d	7.23 d
H-11	6.59 dd	6.62 dd
H-12	6.66 d	6.65 d
H-14	6.38d	6.38 d
H-15	6.65 dd	6.65 dd
CH_3-16	0.855 s	0.851 s
CH_3-17	1.193 s	1.189 s
CH_3-18	1.352 s	1.349 s
CH_3-19	1.979 s	1.976 s
CH_3-20	1.994 s	1.990 s
H-2’ ax	1.45 dd	1.46 dd
H-2’ eq	1.84 ddd	1.84 ddd
H-3’	3.99 m	3.99 dddd
H-4’ ax	2.09 dd	2.07 dd
H-4’ eq	2.43 ddd	2.43 ddd
H-10’	6.46 d	6.46 d
H-11’	6.54 dd	6.54 dd
H-12’	6.36 d	6.36 d
H-14’	6.29 d	6.28 d
H-15’	6.70 dd	6.70 dd
CH_3-16’ ax	1.150 s	1.146 s
CH_3-17’ eq	1.205 s	1.202 s
CH_3-18’	1.928 s	1.925 s
CH_3-19’	2.016 s	2.014 s
CH_3-20’	1.994 s	1.976 s

s, singlet; d, doublet; m, multiplet

Table S3. Data collection and refinement statistics.

**Dataset**		Native
**Crystal parameters**		
Space group		$P2_1$
Unit cell dimensions	a, b, c (Å)	73.75, 101.38, 112.59
	β (°)	95.06
**Data collection**		
Wavelength (Å)		1.0000
Resolution range (Å)[a]		75.21–2.44 (2.50–2.44)
Total reflections[a]		200,767 (14,304)
Unique reflections[a]		61,509 (4,535)
R_{merge}[a, b]		0.104 (0.584)
R_{meas}[a, c]		0.125 (0.702)
R_{pim}[a, d]		0.068 (0.385)
$CC_{1/2}$[a, e]		0.981 (0.746)
Average $I/\sigma(I)$[a, f]		7.0 (1.8)
Completeness[a]		0.999 (0.997)
Redundancy[a]		3.3 (3.2)
**Refinement**		
Resolution range (Å)[a]		75.32–2.44 (2.50–2.44)
R_{work}/R_{free} R-factor[a, g, h]		0.203 (0.317)/0.253 (0.348)
Reflections used for refinement		58,532
Non-hydrogen atoms in an asymmetric unit		
Protein		10,995
Ligand		352
Water		260
Average B-factors (Å²)		
Protein/N-glycans		46.1
Ligand		37.1
Water		39.8
Deviations from ideal geometry		
Bond distances (Å)		0.007
Bond angles (°)		1.471
Ramachandran plot		
Favored (%)		98.6
Allowed (%)		1.4
Outlier (%)		0.0

[a]Values in parentheses are for the highest resolution shell.

[b]$R_{merge} = \Sigma_{hkl}\Sigma_i(|I_i(hkl) - \langle I(hkl)\rangle|)/\Sigma_{hkl}\Sigma_i I_i(hkl)$.

[c]$R_{meas} = \Sigma_{hkl}[N/(N-1)]^{1/2}\Sigma_i(|I_i(hkl) - \langle I(hkl)\rangle|)/\Sigma_{hkl}\Sigma_i I_i(hkl)$.

[d]$R_{pim} = \Sigma_{hkl}[1/(N-1)]^{1/2}\Sigma_i |I_i(hkl) - \langle I(hkl)\rangle|/\Sigma_{hkl}\Sigma_i I_i(hkl)$.

[e]$CC_{1/2}$ is a correlation coefficient between intensities from random half datasets.

[f]Signal-to-noise ratio of intensities.

[g]$R = \Sigma(|F_o - F_c|)/\Sigma F_o$.

[h]Five percent of reflections were randomly chosen for calculating the free R-factor.

Table S4. Information of proteins used for phylogenetic analysis in Fig. 6*A*.

Organism*	UniprotKB ID /accession No.	EPD subgroup
Anolis carolinensis (Green anole)	G1KU93	MERP/EPDR1/EPDR1-like
Chelonia mydas (Green sea-turtle)	M7B3T2	MERP/EPDR1/EPDR1-like
Crassostrea gigas (Pacific oyster)	K1QAN0	MERP/EPDR1/EPDR1-like
Gallus gallus (Chicken)	A0A1D5PC09	MERP/EPDR1/EPDR1-like
Homo sapiens (Human)	Q9UM22	MERP/EPDR1/EPDR1-like
Latimeria chalumnae (West Indian ocean coelacanth)	H3AA94	MERP/EPDR1/EPDR1-like
Lepisosteus oculatus (Spotted gar)	W5N096	MERP/EPDR1/EPDR1-like
Lottia gigantea (Giant owl limpet)	V4CIC5	MERP/EPDR1/EPDR1-like
Monodelphis domestica (Gray short-tailed opossum)	F6PI86	MERP/EPDR1/EPDR1-like
Mus musculus (Mouse)	Q99M71	MERP/EPDR1/EPDR1-like
Xenopus tropicalis (Western clawed frog)	F6VRB7	MERP/EPDR1/EPDR1-like
Danio rerio (Zebrafish)	P17561	Fish-specific / Ependymin/Ependymin-like
Oryzias latipes (Japanese rice fish)	H2MWK0	Fish-specific / Ependymin/Ependymin-like
Salmo salar (Atlantic salmon)	A0A1S3L2Y0	Fish-specific / Ependymin/Ependymin-like
Takifugu rubripes (Japanese pufferfish)	H2U0P6	Fish-specific / Ependymin/Ependymin-like
Ciona intestinalis (Transparent sea squirt)	F6T7K5	Basal-1
Leishmania major (Trypanosomatida)	E9AFK1	Basal-1
Naegleria gruberi (Amoeba)	D2VWS8	Basal-1
Amphimedon queenslandica (Sponge)	I1E8N0	Basal-2
Capsaspora owczarzaki (Amoeba-like Filasterea)	A0A0D2WJ01	Basal-2
Ciona intestinalis (Transparent sea squirt)	F6YPW2	Basal-2
Anopheles gambiae (African malaria mosquito)	Q7QL68	EPDR+cathepsin
Anolis carolinensis-2 (Green anole)	H9G4N8	EPDR+cathepsin
Branchiostoma floridae (Florida lancelet)	C3YBJ4	EPDR+cathepsin
Chlamydomonas reinhardtii (Microalgae)	A8IGP7	EPDR+cathepsin
Dictyostelium discoideum (Slime mold)	Q54TR1	EPDR+cathepsin
Drosophila melanogaster (Fruit fly)	Q9V3U6	EPDR+cathepsin
Nematostella vectensis (Sea anemone)	A7SM85	EPDR+cathepsin
Trichomonas vaginalis (Flagellated protist)	A2ECZ7	EPDR+cathepsin
Trichoplax adhaerens (Amoeba-like placozoan)	B3RJ92	EPDR+cathepsin
Arabidopsis thaliana (Mouse-ear cress)	Q8L9Q7	Plant-specific
Zea mays (Maize)	A0A1D6LBY3	Plant-specific
EPD-BCP1alpha (Blue sponge)	LC494533	This study
EPD-BCP1beta (Blue sponge)	LC737963	This study

* Protein sequences were obtained from a previous study (27).

Supplementary references

1. Englert, G. (1995) NMR Spectroscopy. *Carotenoids: Spectroscopy*, eds Britton G, Liaaen-Jensen S, Pfander H. (Birkhäuser Verlag, Basel), vol. 1B, pp. 147-260
2. Maoka, T. & Fujiwara, Y. (1996) Absolute configurations of mytiloxanthin and 9-E-mytiloxanthin. *J. Japan Oil Chem. Soc.* **45**, 667–670
